# Enhancing blood transfusion safety through RFID-based verification and human factors engineering

**DOI:** 10.3389/fmedt.2026.1899583

**Published:** 2026-08-27

**Authors:** Alessandra Angelucci, Fabiana Zummo, Riccardo Giudici, Roberto Fumagalli, Andrea Aliverti, Gabriele Bassi

**Affiliations:** 1 Dipartimento di Elettronica, Informazione e Bioingegneria, Politecnico di Milano, Milan, Italy; 2 Department of Anaesthesia and Critical Care, ASST Grande Ospedale Metropolitano Niguarda, Milan, Italy; 3 School of Medicine and Surgery, University of Milan-Bicocca, Milan, Italy

**Keywords:** blood transfusion error, human factors engineering, radio frequency identification, RFID, tablet app

## Abstract

**Introduction:**

Blood transfusion is a high-risk process, with bedside verification being the last opportunity to intercept potentially fatal errors. This study presents the design, development, and evaluation of an RFID-based system for bedside transfusion verification, grounded in Human Factors Engineering principles.

**Methods:**

The system employs passive UHF RFID tags on patients, blood units, and healthcare staff to perform continuous silent verification. When all the needed elements for transfusion (patient, matching blood unit, two healthcare operators) are detected by an active RFID reader and no other possible sources of error are present (e.g., another blood unit or patient nearby), the two operators can proceed with a digital checklist. Completion of the digital checklist by the two operators is enforced before transfusion starts. The system was evaluated in simulated scenarios through a series of technical and procedural tests. The development process was informed by iterative feedback from clinical and technical stakeholders, including nurses, critical care physicians specialized in transfusion medicine, and biomedical engineers.

**Results:**

Low detection latency (<300 ms), strong Bluetooth Low Energy stability, sufficient power autonomy (>8 h), and consistent alert behavior under error scenarios were confirmed. Environmental interference from liquids and metal reduced tag readability beyond 130 cm, highlighting positioning and presence of obstacles as a deployment consideration.

**Discussion:**

A limitation of the system is that it does not close the loop, i.e., there is no flow sensor that confirms if infusion is taking place and lasts as long as expected. Still, the system presents a significant advantage with respect to existing checklists and barcode-based systems, as it performs passive checks and is silent when no source of error is identified. The proposed RFID-based workflow showed technical feasibility and correct functional behavior in predefined simulated transfusion scenarios, supporting its potential as an additional safety layer to be evaluated in future clinical studies. No real-world clinical testing was performed in this study; therefore, the present work should be interpreted as a pre-clinical proof-of-concept. Future studies should evaluate the system in actual transfusion settings and compare it with current standard verification practices.

## 1 Introduction

Blood transfusion is a routine procedure employed in several clinical contexts such as surgery, oncology, hematology, and intensive care. In Italy, over 2.8 million blood units were transfused in 2023, corresponding to more than 7,700 average daily transfusions (1). Among all phases of the transfusion process (blood unit collection, compatibility testing, logistic organization to deliver the right blood unit to the ward, and administration of the blood unit), the final administration step at the patient's bedside is a source of residual risk.

Among transfusion-related adverse events, errors of patient or unit misidentification, which is called Incorrect Blood Component Transfused (IBCT), are among the most harmful. In 2023, 11 IBCT events were reported in Italy, of which 84.6% were attributed to patient identification errors, confirming that bedside verification is a critical vulnerability (1). As several near misses, *i.e.*, errors that were found and corrected before the wrong transfusion happened, go unreported, figures on bedside identification error are likely underestimated (2). Studies in Japan (3), the United Kingdom (4), and the United States (5) have consistently shown that human error at the patient's bedside, particularly related to patient identification, is the leading cause of ABO-incompatible transfusions, *i.e.*, the administration of a blood unit with a mismatched blood group to a patient. Such events can cause severe immune reactions leading to red blood cell destruction (hemolysis), kidney damage, coagulation disorders, and can be fatal.

The transfusion process can be summarized into four phases: pre-transfusion sampling; immunohematological testing to assess compatibility between donor and recipient; delivery of the blood unit from the laboratory to the ward where it will be administered; administration of the blood unit to the patient. It is during the last phase, at the patient's bedside, that final verification takes place. Operators must check multiple identifiers: patient name, date of birth, unit number, blood group, and clinical documentation.

Italian guidelines mandate a double-check procedure performed by two healthcare workers (6), which is done with a paper-based checklist to be compiled at the patient's bedside. Such checklists have been proven to reduce errors and consequent outcomes in multiple settings, among which surgery, also because of the behavioral change requested to operators to perform the checklist itself (7). However, such checklists are also susceptible to error, omission, or procedural bypass in practice, especially in high-stress environments such as intensive care (8). In many healthcare systems, particularly in the United States, this verification process is supported by electronic health record (EHR) systems. For example, the Blood Product Administration Module (BPAM) implemented in the EPIC EHR enforces electronic verification of patient identity and blood unit compatibility at the bedside (9). These systems require confirmation by two healthcare professionals and are designed to prevent data omission and reduce the risk of transfusion errors by enforcing structured workflows and real-time validation.

Human Factors Engineering (HFE), also known as ergonomics, offers a system-level framework for analyzing and mitigating errors that are not due to lack of knowledge nor procedures. Originating in aviation and nuclear safety, HFE is increasingly recognized as essential in healthcare, particularly in high-risk procedures such as surgery, medication administration, and transfusion medicine (10). The Institute of Medicine's report *To Err is Human* emphasized that safety must be “designed into” processes from the outset, rather than retrofitted through compliance enforcement (11).

HFE views the healthcare system as a socio-technical environment composed of people, technologies, workflows, and organizational rules. Applying HFE means designing tools and procedures that align with human cognitive and physical limitations, thereby making the “right thing easy to do and the wrong thing hard to do”. In the context of transfusion, this translates into reducing reliance on memory, enforcing procedural compliance without increasing cognitive burden, and building safety into the infrastructure of bedside verification.

To address the limitations of bedside verification, some hospitals have adopted technological solutions such as barcode scanning systems (2). Blood component labeling is standardized according to the International Society of Blood Transfusion (ISBT) specifications, which rely on barcode-based identification to ensure traceability and interoperability across systems. Electronic verification systems such as BPAM are explicitly designed to scan these standardized barcodes, enabling accurate matching between patient identity and blood unit at the bedside within a regulated workflow. These systems typically require scanning a label on the patient's wristband and the label on the blood unit to verify identity, matching and compatibility. A case study by Chou et al. (12) showed a significant reduction in transfusion errors (from 0.03% in 2008–2010 to 0.002% in 2016 and then to 0.001% in 2017) after introducing a barcode-based verification system. The study by Nuttall et al. (13) at Mayo Clinic showed that the implementation of a barcode-based verification system was also associated with a substantial increase in detection of near-misses.

However, barcode systems have limitations. To check whether barcodes are corresponding, healthcare operators need to use a dedicated scanner (which might not be immediately available to operators as they constantly move across the ward), align the scanner to the label, and scan within the line-of-sight. There is also the possibility that the detector does not work properly: this could happen because of environmental constraints which prevent the operators from performing the procedure perfectly, or because the label itself is ruined or damaged, among other possible events. All these constraints can lead to procedural workarounds or scanning omissions, especially under emergency or time-sensitive conditions.

Radio Frequency Identification (RFID) are a promising alternative. Unlike barcodes, RFID tags do not need line-of-sight nor specific alignment to be detected by an active reader, and readers can simultaneously detect multiple items within a defined space. In transfusion medicine, high frequency (HF) RFID tags are already used in the blood supply chain to track units during production and storage (14). Ultra-high frequency (UHF) RFID tags have been also already proposed in the literature for the same purpose (15).

Despite these advantages, the adoption of RFID technologies across the entire blood supply chain is still limited, in part due to higher costs compared to barcode-based systems, particularly when considering large-scale deployment and infrastructure requirements (16).

This work presents the design, development, and testing of a silent RFID-based verification system for bedside transfusion error prevention, based on HFE principles. Technical and simulated bedside tests were conducted to evaluate performance, and correctness of the output in different conditions. The objective of this work was to design and develop a passive RFID-based bedside verification prototype, informed by HFE principles, and to evaluate, in a controlled pre-clinical setting, its technical feasibility, functional behavior, and ability to detect predefined correct and unsafe configurations during simulated transfusion workflows.

## 2 Materials and methodsn

We designed, developed, and evaluated a system that integrates passive RFID technology with HFE principles. The system was conceived as a safety net, running silently in the background and intervening only in the presence of risks. Given the early-stage nature of the prototype, the present evaluation was intentionally limited to technical testing and simulated transfusion scenarios in a controlled environment. No testing involving patients or routine clinical care was performed.

### 2.1 System overview

The solution is composed of three main components: a wireless UHF RFID reader paired to an Android tablet via Bluetooth Low Energy (BLE), and passive RFID tags applied to blood bags, patients, and healthcare operators. UHF RFID was selected because it provides a substantially longer read range than both barcode systems and conventional HF RFID. HF tags require very close proximity (typically a few centimeters), making continuous verification impossible in the context of blood transfusions, whereas UHF tags can be detected passively at distances up to a few meters in ideal conditions. This extended range enables automatic, simultaneous detection of the patient, the prescribed blood unit, and both operators without requiring deliberate scans. Compared with barcode-based workflows, which depend on manual line-of-sight scanning and are prone to omission in time-pressured environments, UHF RFID supports a fully passive verification layer that cannot be bypassed and does not increase operator workload.

**Figure 1:**
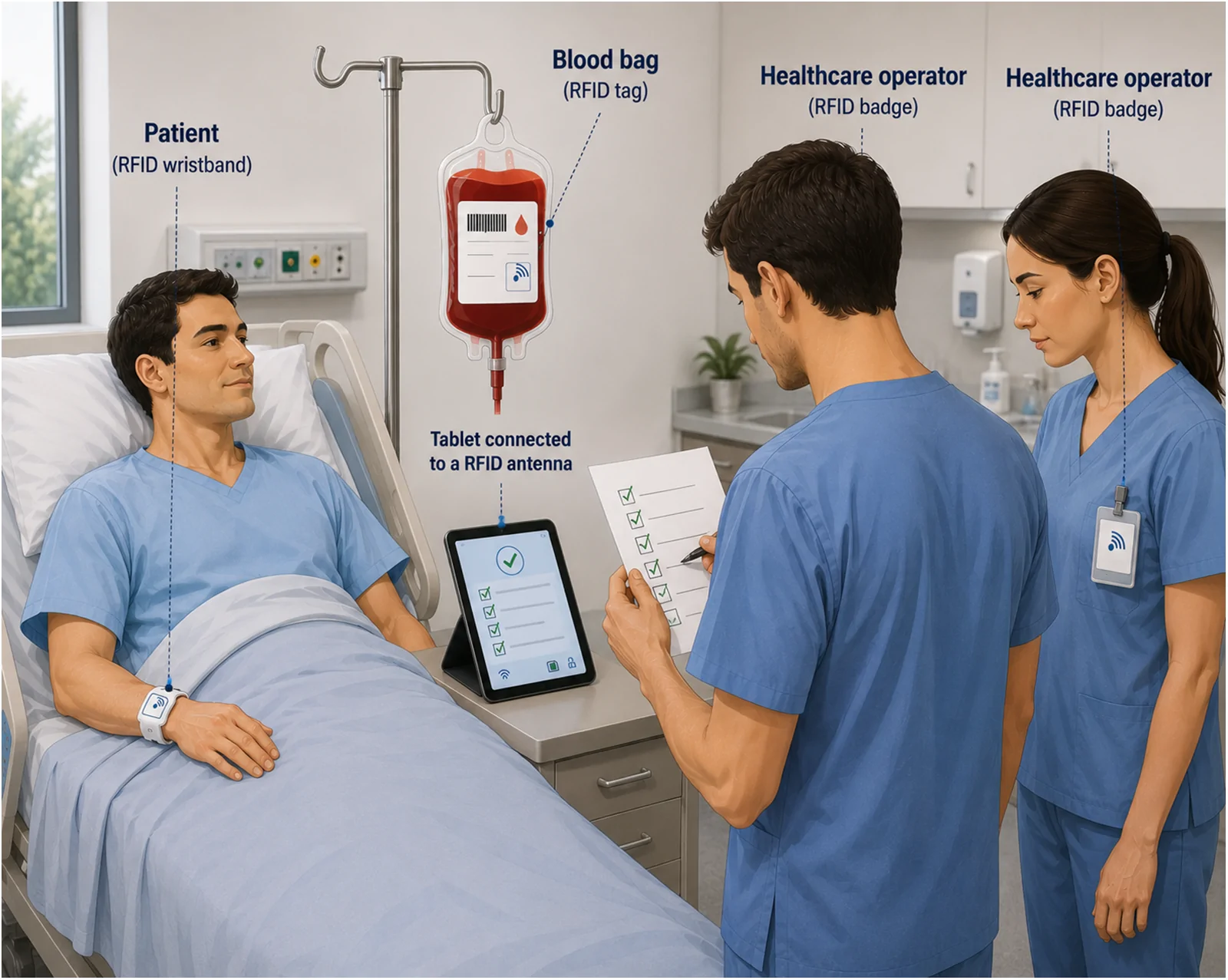
Presence of RFID reader and tags in a blood transfusion scenario: a blood bag matching with the patient is transfused with two healthcare operators present, as requested by Italian regulations.

Figure 1 illustrates a schematic of the system working in ideal conditions.

The skID reader (CAEN RFID S.r.l., Viareggio, Italy) works in the UHF band (865–868 MHz with up to 500 mW of RF power, EPC Gen2 compliant), with BLE used to communicate with the tablet. The UHF RFID reader is a compact stand-alone device that communicates with the tablet via BLE. During use, it is typically placed adjacent to or resting against the tablet for ease of handling, but it can also be positioned at short distance from the tablet (up to several centimeters) without affecting communication. The reader is therefore not mechanically integrated with the tablet, and its placement at the bedside can be adjusted depending on clinical constraints. Patients and blood bags are each associated to a passive UHF RFID tag (EPC Gen2; ISO 18000-63 compliant) with a unique identifier.

The Android application was developed in Kotlin and built to function autonomously once launched: it scans the environment continuously, detects the presence of the expected elements, and only enables to access the next phases of the workflow (*i.e.*, checklist after scanning, start transfusion after checklist) when the proper configuration of RFID tags is verified.

The system is designed to operate in compliance with existing transfusion safety workflows and is intended to complement, rather than replace, regulated identification procedures.

### 2.2 Operational workflow

When the tablet application is started, the RFID reader, paired to the tablet application via BLE, begins scanning the environment in real time, searching for one patient tag, one blood unit tag (paired to the patient), and two healthcare operator tags. The presence of such tags and no other possible source of error are necessary conditions for allowing the transfusion to go ahead. It must be noted that the current implementation is designed to support transfusion scenarios involving a single blood unit at a time. This choice reflects the intended use in controlled clinical environments such as hospital wards or outpatient settings, where transfusions are typically administered sequentially rather than concurrently. Scenarios involving multiple simultaneous blood units, such as in operating rooms or emergency settings, are outside the scope of the present system and represent an area for future development.

Once all required tags are simultaneously present within the field of detection and correctly matched according to the database of valid pairings (*i.e.*, the blood bag is indeed prescribed to the patient), the application transitions to the next phase. In the current prototype, this matching is performed using a local database of predefined patient–blood unit associations. In a real clinical deployment, this information would be retrieved from hospital information systems, such as the Blood Bank Laboratory Information System (LIS), which is responsible for assigning compatible blood units to patients following immunohematological testing. The local encrypted database is intended as a secure application-level audit trail and does not replace the patient's official medical record. In a real clinical deployment, the relevant data captured by the system should be transferred to and documented within the hospital EHR/patient chart and integrated with transfusion information systems (*e.g.*, LIS), so that transfusion events are incorporated into the formal clinical documentation according to local regulatory requirements.

In this work, the term “silent check” refers to the normal verification mode of the system: RFID detection and matching are performed continuously in the background, but no notification is generated as long as the expected configuration is maintained. Alerts are displayed only when the system detects an abnormal or potentially unsafe configuration. Red alerts are triggered by mismatches (*e.g.*, ABO incompatibility), presence of more tags than necessary (*e.g.*, more than one blood bag detected, more than one patient detected), or missing tags. Technical tests, which will be discussed later, showed that, in realistic clinical environments with liquid and metal interference, stable read range decreases to approximately 110–130 cm, with signal loss beyond this distance. This natural attenuation, combined with the exclusion logic, ensures that the system does not proceed when tags from patients in neighboring beds are inadvertently detected, thereby avoiding misidentification. Yellow alerts are triggered by anomalies (*e.g.*, the blood bag removed or signal loss). Both red and yellow alerts prevent the operators from starting or completing the checklist. In the current prototype, yellow alerts were implemented as visual warnings only, whereas red alerts were implemented as blocking alerts and may be associated with an acoustic notification depending on the configuration. When the status color is green, only correct elements are present, allowing the checklist to be accessed.

This checklist includes all the elements present in the guidelines of the Italian Ministerial Decree of November 2, 2015 (Annex 7) (6), which defines the mandatory bedside verification steps for transfusion. In particular, the checklist requires confirmation of patient identity (e.g., name and date of birth, with active patient involvement when possible), verification of blood group compatibility, and validation of blood unit information, including identification code and expiry date.

While the current implementation reflects the Italian regulatory framework, the checklist structure is configurable and can be adapted to other national or institutional requirements. For example, additional identifiers such as medical record number or unique donation identification number (DIN) can be incorporated to comply with different safety standards.

If at any point a mismatch is detected, because of an incompatible blood unit, multiple components in the environment, or the absence of one of the healthcare operators, the system triggers an alert, and the checklist is locked, preventing the transfusion from going ahead until the safety condition is resolved and only correct RFID tags are present nearby. To prevent signal conflicts between patients in adjacent beds, the system applies a conservative multi-tag exclusion rule: the detection of more than one patient tag or more than one blood-unit tag automatically blocks progression and triggers an alert.

All actions, including tag detections, checklist interactions, alert triggers, and operator confirmations, are time-stamped and stored in a local encrypted database. At the end of the procedure, the application generates a digitally signed PDF report that includes all relevant metadata, including a history of all RFID tags that have been detected from the beginning of the scanning to the completion of the transfusion, thus ensuring traceability of events and identification of near misses.

**Figure 2:**
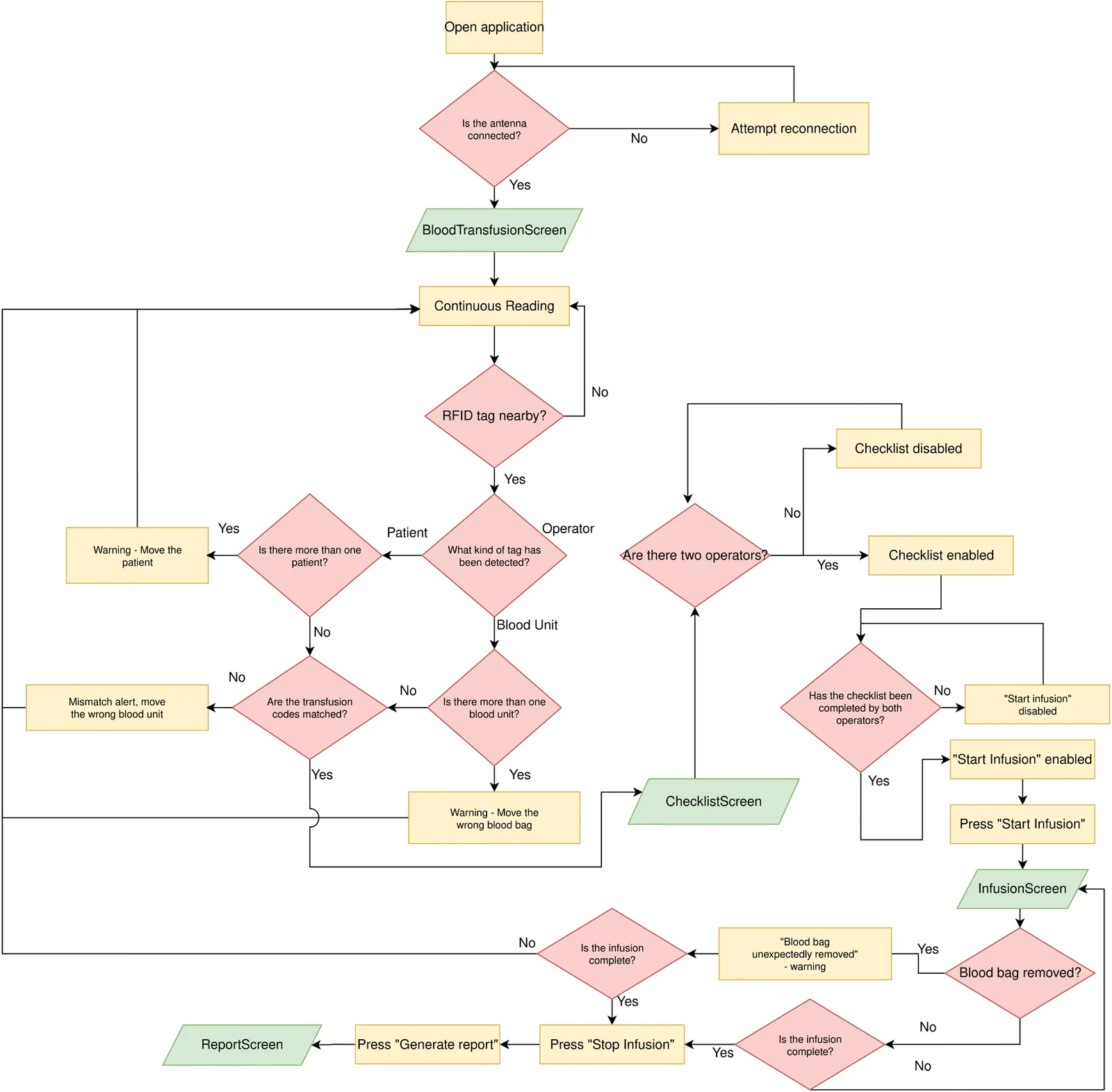
Flowchart of the application.

Figure 2 illustrates the flowchart of the application and details the transitions between different system states (17).

### 2.3 Logic and technical testing

A series of structured tests were conducted in a controlled simulation environment. These included technical evaluations of the hardware and software components, and simulated transfusion scenarios reproducing realistic hospital workflows.

Alert logic was assessed first with the correct procedure and then through simulated error scenarios, including introduction of a second (unmatched) blood bag, attempted transfusion with a mismatched unit, absence of a second operator, tag removal during the infusion procedure.

System responsiveness was measured by manually recording the latency from tag introduction to user interface confirmation. This was performed with a chronometer and synchronized video analysis to ensure measurement accuracy.

Power autonomy was assessed by running the system continuously at full charge.

Bluetooth stability was assessed at increasing distances between the reader and tablet.

Tag reading performance was evaluated by measuring detection range under four conditions: A) clean, interference-free environment; B) simulated hospital bed with metal surfaces; C) proximity to liquid-filled containers (to simulate blood bags); D) presence of metallic structures like IV poles and trays. For each condition, distance was increased in 20 cm steps and tag visibility was classified as valid, unstable, or null. The same tag model was used throughout all scenarios to ensure comparability.

Each technical test and simulated scenario was performed once under the previously described predefined controlled conditions; therefore, the evaluation was intended to verify expected system behavior rather than to estimate statistical performance metrics.

## 3 Results

This section reports the results of the different tests performed to evaluate the functioning of the solution (Figure 1), in terms of correct output in the different scenarios and technical performance.

### 3.1 Alert logic

**Figure 3:**
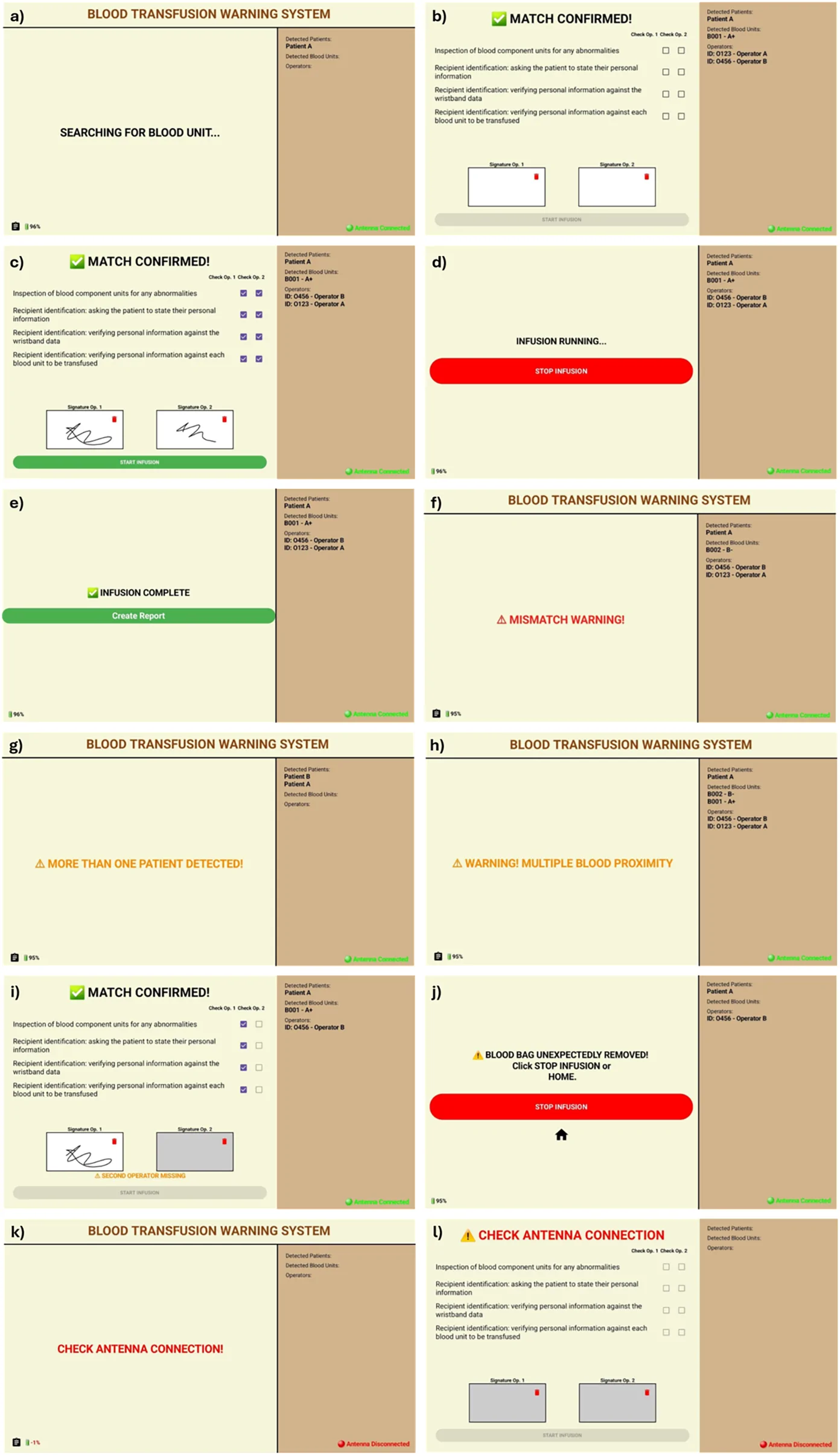
Tablet app and warnings. **(a)** Searching screen, only one patient detected; **(b)** Checklist screen, available only if all RFID tags match and reflecting mandatory bedside verification steps defined by national transfusion guidelines; **(c)** Completed checklist screen, which allows to start the transfusion; **(d)** Transfusion procedure running screen, with possibility to stop; **(e)** Completed transfusion procedure screen and possibility to create a final report; **(f)** Red alert: mismatch between patient and blood unit detected; **(g)** Yellow alert: more than one patient detected in proximity of the reader; **(h)** Yellow alert: more than one blood unit detected in proximity of the reader; **(i)** Yellow alert: second operator missing during checklist completion; **(j)** Yellow alert: blood bag not detected anymore after transfusion started; **(k)** Red alert: antenna disconnected during passive tag search phase; **(l)** Red alert: antenna disconnected during checklist.

All the tested conditions to verify the functioning of the alert logic are reported in Figure 3. The *BloodTransfusionScreen* in the flowchart (Figure 2) dynamically lists the detected tags (Figure 3a). Upon successful verification, the app transitions to the *ChecklistScreen* of the flowchart, which requires both operators to confirm the four verification items via checkboxes and digital signatures. Figures 3b,c show this screen before and after completion. When the checklist is completed, the transfusion can start. When the transfusion is running, there is the possibility to manually stop it (Figure 3d). When it is completed, a PDF report is generated (Figure 3e).

A mismatch between patient and blood bag triggers an immediate visual (Figure 3f) and acoustic alert, and the checklist cannot be accessed. The presence of more than one patient triggers an alert and prevents operators from going ahead (Figure 3g). Also, when two or more blood bags are detected within the reading field, the system displays a specific alert and blocks checklist access (Figure 3h).

As the checklist requires dual verification, if only one healthcare worker is present, the application locks the second column of the checklist, preventing initiation of the infusion (Figure 3i). The system raises an immediate warning if the blood bag is removed before the manual confirmation of transfusion completion (Figure 3j). Finally, there is the disconnection between the tablet application and the antenna, both during the tag searching phase (Figure 3k) and the checklist completion phase (Figure 3l).

### 3.2 Technical testing

In terms of power autonomy during continuous use, the RFID reader remained operational for approximately 8.3 h before shutting down, while the tablet still had 34% battery. The system can therefore work for a full shift (8 h). As the antenna is wireless and connected via BLE, it can also be charged while still functioning.

In all trials, the latency between tag exposure and visualization on the tablet interface was consistently under 300 ms, which is essentially instantaneous from a clinical perspective.

Stable BLE connection between the RFID reader and the tablet in a controlled clinical environment was obtained up to 625 cm, covering all expected bedside configurations.

**Figure 4:**
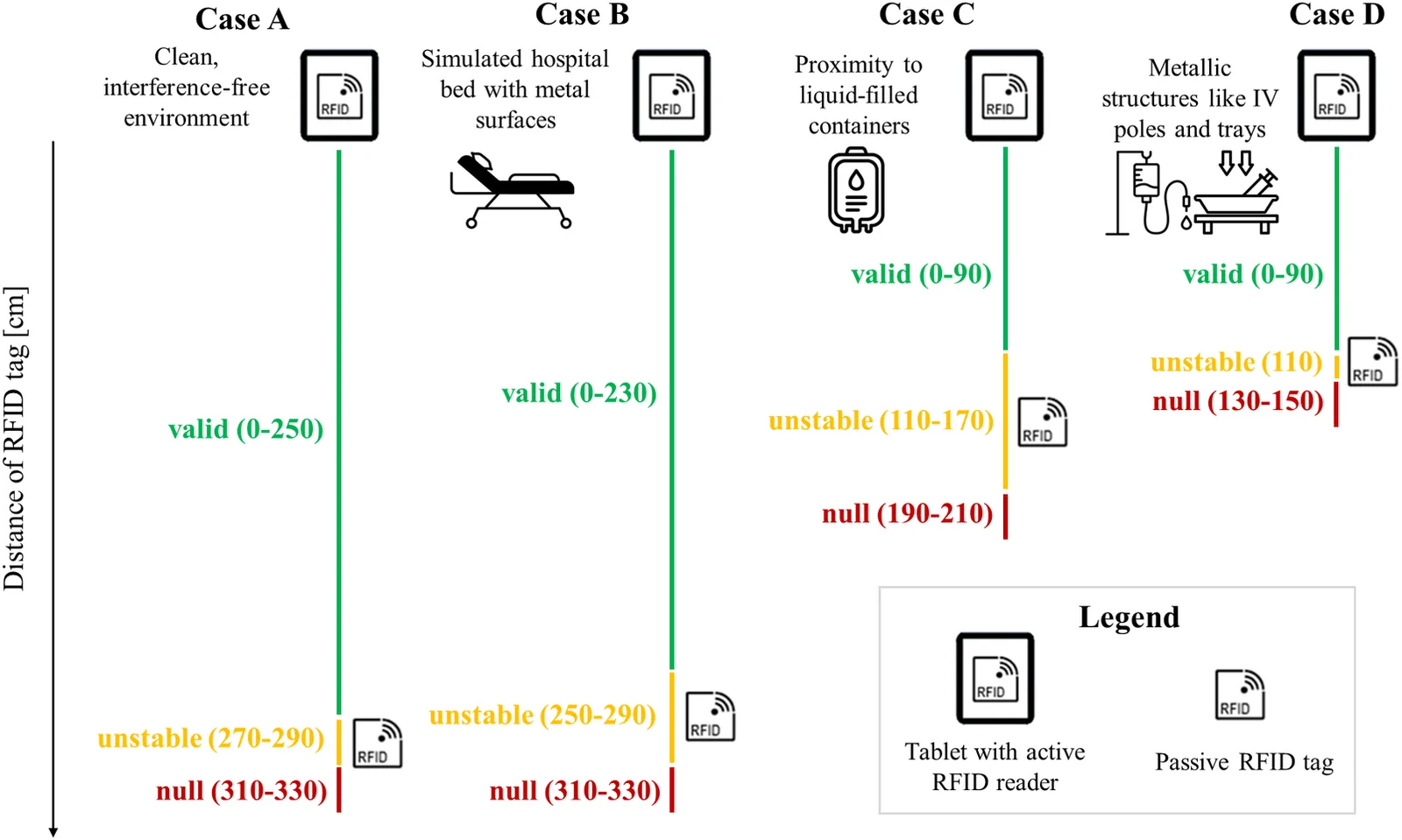
Tag Reading performances in different testing conditions: **(A)** clean, interference-free environment; **(B)** simulated hospital bed with metal surfaces; **(C)** proximity to liquid-filled containers (to simulate blood bags); **(D)** presence of metallic structures like IV Poles and trays.

Finally, tag reading performance in all tested conditions is reported in Figure 4. Tag visibility was scored as *valid* (stable detection), *unstable* (flickering), or *null* (no detection). The maximum distance for reliable detection in a clean environment reached 250 cm (case A). Performance dropped significantly when liquid or metallic interference was introduced (cases B, C, D), which is representative of conditions in hospital wards.

## 4 Discussion

Errors in bedside transfusion administration are an issue, even in settings with elevated levels of standardization in the earlier phases of the transfusion chain. The solution presented in this study directly addresses this gap by combining RFID-based verification with a checklist-enforcing logic, aligned with the principles of HFE.

Previous applications of RFID in healthcare have primarily focused on supply-chain tracking or inventory management, whereas bedside verification workflows typically rely on barcode scanning. The present system differs in that it applies UHF RFID to enable continuous, passive, multi-tag verification of patient, operator, and blood-unit presence, integrated with enforced digital checklist logic. To our knowledge, no published system combines UHF passive detection with procedural enforcement designed with HFE principles in mind.

By design, the system enforces verification passively. RFID tags on the patient, blood unit, and operators are detected without requiring line-of-sight or manual scans. Barcode systems, which have proven effective in detecting both errors and near misses (12, 13), rely on scanning steps that may be skipped or poorly executed in real-world settings. In such circumstances, safety is maintained through regulated manual or downtime verification procedures, typically requiring dual-operator confirmation. The passive nature of RFID tags and the silent checks performed by the RFID reader may further reduce the risk of omission of procedural steps by adding a continuous automated verification layer.

Furthermore, the system ensures that transfusions can only proceed after compliance with a digital checklist, whose contents are decided at the national level (6). The checklist is not merely digitized but procedurally enforced, becoming accessible only when all safety conditions are confirmed. This drops the risk of omission of essential checks (18). In fact, checklist fatigue is a known problem in the healthcare context, even if the benefits of using checklists in terms of safety and reduction of errors have been largely demonstrated (19); for this reason enforcing the procedure is critical. Importantly, current transfusion practices are governed by strict regulatory frameworks (e.g., FDA in the United States, European and national guidelines), which require robust identification of the right patient and the right blood unit, typically including verification by two healthcare workers. In the proposed system, this safety logic is extended to the simultaneous presence of the intended patient, the correctly matched blood unit, and two healthcare operators identified through their RFID tags. However, the system is designed to operate within existing regulated workflows as an additional safety layer, rather than as a replacement for mandated identification procedures. Even in settings where barcode-based electronic workflows are unavailable, interrupted, or bypassed, transfusion practice still relies on regulated manual or downtime documentation procedures requiring dual verification; our system is intended to support and strengthen these checks, not substitute for them.

The tablet application and backend were designed by respecting the principles of HFE, which promote systems that make the right action easy and the wrong action difficult or impossible impossible (11). However, the development process should not be interpreted as a formal participatory design study (20). Rather, the prototype was developed through an expert-informed process, in which input from clinical and technical stakeholders (nurses, critical care physicians specialized in transfusion medicine, and biomedical engineers) was considered during the definition of the workflow, alert logic, and checklist implementation. These inputs helped align the prototype with bedside transfusion practices and clinical constraints, although no predefined participant selection strategy, structured qualitative analysis, or formal usability assessment was performed. Consistent with this design rationale, the system was conceived to minimize unnecessary interruptions: checks are performed silently in the background, and alerts are displayed only when a potentially unsafe configuration is detected. This choice is particularly relevant in intensive care and other high-pressure environments, where alert fatigue may reduce the effectiveness of safety systems (21). Therefore, although the system was developed according to HFE principles, the human factors contribution of this study should be interpreted primarily at the design level rather than as a validated demonstration of usability, reduced operator burden, or improved operator acceptance.

The technical evaluation confirmed reliable detection, low latency, and acceptable power autonomy. However, changing environmental conditions have a measurable effect on tag readability and performance. Presence of liquid containers (*e.g.,* saline, blood bags) and metallic objects (*e.g.*, IV poles) reduced tag detection range, with performance dropping from 250 cm in ideal conditions to <130 cm in setups with different objects, with the latter cases being more representative of actual clinical applications. While the core functionality is still intact in different measurement conditions, proper antenna placement and user training will be essential for reliable field performance.

In addition to technical constraints, economic considerations represent a relevant limitation. Compared to barcode-based systems, RFID technology involves higher costs related to active readers and system integration. While passive RFID tags are relatively low-cost and are purchased in bulk, large-scale implementation across the entire transfusion chain may still represent a significant investment.

A core limitation of the current prototype is that it is not a closed-loop system. It verifies set-up and continuity (*i.e.,* that the correct tags are still present during infusion), but does not assess whether blood was infused. Adding a flow sensor for real-time monitoring of intravenous infusion (22) could address this gap, further improving safety.

A critical aspect for real-world deployment of the proposed system is the correct association between RFID tags and their corresponding entities (patients, blood units, and healthcare operators). In a clinical implementation, tag assignment should not be treated as an informal upstream assumption, but should be designed as a controlled safety-critical step integrated into existing identification and transfusion workflows. For patients, RFID tag pairing could be performed at admission or at the time of wristband application, with barcode cross-verification against the existing patient wristband and EHR record. For blood units, RFID assignments should be embedded in blood bank preparation and release procedures, with automatic linkage to the unit identifier through the Blood Bank LIS or transfusion management system. For healthcare operators, RFID badges should be linked to institutional staff identity-management systems.

For high-risk associations, particularly patient and blood-unit pairing, an enforced verification step should be included, such as dual-operator sign-off or equivalent electronic confirmation. Embedding RFID tag assignment into existing regulated workflows would reduce the risk of upstream identification errors propagating into the RFID-based bedside verification layer.

Electronic verification systems integrated into EHR platforms, such as the BPAM in EPIC, represent a widely adopted approach to transfusion safety, particularly in the United States. These systems are built upon ISBT-standardized barcode labeling of blood components and enforce structured workflows in which barcode scanning is used to confirm correct patient–blood unit matching at the bedside. Barcode-based solutions have shown reductions in transfusion errors, with some studies reporting near-elimination of mismatches (12). However, these systems depend on operator discipline and adherence to the procedure, which involves an added active task by the operators. If the procedure is bypassed for any reason, errors are not intercepted. While BPAM and similar systems rely on active user interaction (*e.g.*, barcode scanning and manual confirmation), RFID enables automatic detection of the patient, blood unit, and operators without requiring explicit scanning actions. This distinction may be particularly relevant in high-workload or time-critical environments, where procedural steps may be delayed or inconsistently executed (14).

No human-subject testing or real-time clinical evaluation was performed in this study. Accordingly, the proposed system should be interpreted as a pre-clinical proof-of-concept. Future investigations will need to evaluate its performance in real transfusion settings and compare it with current standard blood transfusion safety practices. Because the present study was designed as a pre-clinical proof-of-concept based on simulated scenarios, formal performance metrics such as sensitivity, specificity, false positive rates, and false negative rates were not estimated. Although the environmental interference tests showed reduced read range in the presence of liquids and metallic obstacles, the study was not powered or structured to quantify diagnostic classification performance under these conditions. The current tests verified expected system behavior in predefined scenarios rather than repeatedly sampling true positive, true negative, false positive, and false negative events. Future clinical validation studies should therefore include repeated trials in realistic bedside settings and quantify sensitivity, specificity, false positive and false negative rates, nuisance alert frequency, and alert reliability across different tag positions, reader orientations, and environmental conditions.

A clinical pilot spanning several weeks will be needed to assess operational robustness, real-world error interception rates, and operator satisfaction. Integration with hospital information systems and EHRs will also be necessary to perform a clinical pilot. In this case, the correct blood patient matching needs to be confirmed by querying the relevant blood bank database (e.g., Blood Bank LIS), or a transfusion management system at the regional and national level depending on the local organization.

Another important aspect for future development is the extension of the system to support scenarios involving multiple simultaneous blood units, which are common in surgical or emergency settings. This would require more advanced logic for managing multiple valid units and ensuring correct association with the patient.

## 5 Conclusions

This study presents a novel bedside transfusion verification system based on RFID and designed through a human-centered approach and based on HFE principles. In simulated settings, the system showed correct behavior across multiple intended and error scenarios, with low latency and adequate technical performance. Its main strengths are passive verification, procedural enforcement, and alignment with clinical workflow requirements. However, the absence of real-world clinical validation and closed-loop infusion confirmation remains a major limitation. Because no clinical outcomes, real-world error rates, usability metrics, or workload measures were assessed, the present findings should not be interpreted as evidence of improved transfusion safety or reduced operator burden. Rather, they demonstrate the technical feasibility and expected behavior of the prototype under controlled simulated conditions.

## Data Availability

The original contributions presented in the study are included in the article/Supplementary Material, further inquiries can be directed to the corresponding author/s.
